# The antidepressant effect of cognitive reappraisal training on individuals cognitively vulnerable to depression: Could cognitive bias be modified through the prefrontal–amygdala circuits?

**DOI:** 10.3389/fnhum.2022.919002

**Published:** 2022-08-04

**Authors:** Xiaoxia Wang, Ying He, Zhengzhi Feng

**Affiliations:** ^1^Department of Basic Psychology, School of Psychology, Army Medical University, Chongqing, China; ^2^Department of Psychiatry, Second Affiliated Hospital, Army Medical University, Chongqing, China; ^3^School of Psychology, Army Medical University, Chongqing, China

**Keywords:** antidepressant effect, cognitive reappraisal training, cognitively vulnerable to depression, prefrontal-amygdala circuit, cognitive bias

## Abstract

Cognitive reappraisal (CR) is one of the core treatment components of cognitive behavioral therapy (CBT) and is the gold standard treatment for major depressive disorders. Accumulating evidence indicates that cognitive reappraisal could function as a protective factor of cognitive vulnerability to depression. However, the neural mechanism by which CR training reduces cognitive vulnerability to depression is unclear. There is ample evidence that the prefrontal–amygdala circuit is involved in CR. This study proposes a novel cognitive bias model of CR training which hypothesizes that CR training may improve the generation ability of CR with altered prefrontal–amygdala functional activation/connectivity, thus reducing negative cognitive bias (negative attention bias, negative memory bias, negative interpretation bias, and/or negative rumination bias) and alleviating depressive symptoms. This study aims to (1) explore whether there is abnormal CR strategy generation ability in individuals who are cognitively vulnerable to depression; (2) test the hypothesis that CR training alleviates depressive symptoms through the mediators of cognitive bias (interpretation bias and/or rumination bias); (3) explore the neural mechanism by which CR training may enhance the ability of CR strategy generation; and (4) examine the short- and long-term effects of CR training on the reduction in depressive symptoms in individuals who are cognitively vulnerable to depression following intervention and 6 months later. The study is promising, providing theoretical and practical evidence for the early intervention of depression-vulnerable individuals.

## Introduction

Depressive disorder is an affective mental disorder with depressive mood and anhedonia as the defining symptoms. It may cause functional disability, seriously affect social functioning and quality of life, and create a heavy socioeconomic burden. The disease burden of depressive disorders accounts for 10.3% of the total disease burden (Smith, 2014), which is estimated to be the cause of the world’s top disease burden in 2030. Therefore, early identification and intervention to reduce disease burden is currently the focus of research on depressive disorders.

According to the cognitive vulnerability–stress theory of depression, cognitive vulnerability can interact with stress to increase the likelihood of depression (Abramson et al., 1989, 1999). Cognitive vulnerability manifests as negative cognitive biases, such as biases in attention, interpretation, and rumination (Dai and Feng, 2008; Chen and Feng, 2015; Zhang and Feng, 2016; Yan et al., 2017). The combined cognitive bias hypothesis in depression proposed that negative attention bias can lead to interpretation bias, which in turn induces rumination and subsequently more depressive symptoms. This hypothesis is supported empirically by path analyses of these variables (attention bias→interpretation bias→rumination→depressive symptoms; Everaert et al., 2012, 2017).

Cognitive reappraisal (CR), which changes the affective regulation process of emotional responses through the reinterpretation of the meaning of emotional stimuli, represents a core technique of cognitive behavioral treatment (CBT), the gold standard psychotherapy of depressive disorder (Shore et al., 2017). Typically, depressed individuals manifest deficits in the habitual use of CR strategies. Compared with other emotion regulation strategies (e.g., emotion expression suppression), deficits in positive CR (the frequency with which individuals interpret stressful life events as positive rather than threatening) are specific factors that can predict depressive symptoms (Garnefski et al., 2005) and positive emotions initially and depression and anxiety symptoms 3–6 months later (Nowlan et al., 2016). In contrast, frequent use of CR may have beneficial effects that protect individuals from depression vulnerability. Frequent use of CR can modify an individual’s interpretation bias, dampen rumination bias, and thus alleviate depression symptoms (attention bias→interpretation bias→CR→rumination→depressive symptoms) (Everaert et al., 2017). CR frequency mediated the relationship between interpretation biases and depressive symptoms in individuals at high familial risk for depression and alleviated the negative impact of interpretation bias on depressive symptoms (interpretation bias→CR→depressive symptoms) (Sfärlea et al., 2021). Depression-resilient individuals tend to use CR to assign a new emotional meaning to stressful events (Knyazev et al., 2017), whereas adolescents with parents having a history of depression (depression-vulnerable individuals) who frequently use CR as an emotion regulation strategy will experience less depression and have higher positive emotions (Kudinova et al., 2018). Specifically, CR was positively linked with the ability to inhibit negative content during a negative affective priming task in individuals with low rumination (Daches and Mor, 2015). Individuals who frequently use CR can better inhibit the interference of distracting negative stimuli (Cohen et al., 2012) and have fewer depressive symptoms in response to unpredictable stress (Troy et al., 2013). The ability to generate a non-negative CR predicts fewer stress responses (lower cortisol levels) when faced with depression-related acute stress (Tsumura et al., 2015). Therefore, the CR ability may have a positive impact on reducing depressive symptoms through inhibition or compensation of negative cognitive bias (e.g., interpretation bias and/or rumination bias) as well as through reduction of physiological arousal.

Notably, previous studies utilizing self-report measures, such as the Emotion Regulation Questionnaire (ERQ) (Gross and John, 2003) and Cognitive Emotion Regulation Questionnaire (CERQ), mainly measure an individual’s tendency, such as frequency (trait) and extent (state), to use CR strategies specified by the developers of the questionnaire in daily life. However, CR tendency reflects typical behavioral patterns rather than capacity for best performance (e.g., CR ability) (Perchtold et al., 2017). Furthermore, self-reported CR tendency is moderately correlated with personality factors such as self-acceptance and environmental mastery (*r* = 0.35 and 0.41) (Gross and John, 2003), which may influence the motivation to use CR strategies and cannot objectively quantify the ability of the individual to generate a CR strategy. In contrast, CR ability, which is also termed reappraisal effectiveness (RE), can be experimentally measured by changes in spontaneous emotion responses after the individual is required to adopt a CR strategy compared to passively viewing emotional stimuli (Troy et al., 2010). A previous study demonstrated that deficits in experimentally measured CR ability are manifested in individuals with mild to moderate symptoms of depression (Zhang et al., 2016), patients in depressive episodes (Compare et al., 2014; Wang et al., 2014; Smoski et al., 2015; Wang et al., 2015), and patients in remission from depression (Ehring et al., 2008), and they can be predictive of depressive symptoms after treatment (Radkovsky et al., 2014).

One other issue of concern for CR ability is that most previous CR tasks required the subjects to generate new CR strategies of emotional stimuli under guidance rather than spontaneously (Khodadadifar et al., 2022), making it impossible to directly measure an individual’s spontaneous tendency to produce and maintain a CR strategy. According to the recent empirical literature, CR ability can be operationalized as either RE or reappraisal inventiveness (RI) (Zeier et al., 2021), with RI independent of RE (Zeier et al., 2020). RI is defined as the flexibility and semantic fluency of individuals in generating new emotional appraisals in response to specific emotion-inducing scenes, which is measured with the Reappraisal Inventiveness Test (RIT) (Weber et al., 2013). Despite the well-acknowledged effectiveness of CRs, the launching of CRs is found to be more difficult due to default preferences in the decision to regulate emotions (Suri et al., 2015). The performance of the RIT task includes RIT-fluency (the number of reappraisals generated) and RIT-flexibility (the number of categories of different reappraisals), which will be transformed into RIT scores. Thus, a higher RIT score represents greater CR abilities to generate abundant and diverse strategies to regulate emotion. Studies found that a greater RI predicts lower chronic stress levels in women and depressive symptoms in men (Perchtold et al., 2017, 2019). Greater RI, especially that which generates alternative (creative) reappraisal strategies, predicts immediate, and long-lasting beneficial effects (< 10 min vs. 3 days) in transforming negative emotion into positive emotion (Wu et al., 2019). Furthermore, the reduced ability of individuals with depressive symptoms to generate a CR strategy was observed (Perchtold et al., 2017). Therefore, the RI index is necessary for objective measurement of CR ability, and the improvement in RI may be beneficial to emotional health.

Intervention studies demonstrated that individuals’ CR ability can be improved through training and could predict changes in depressive symptoms. CR training could alleviate depressive symptoms and negative emotions, improve happiness and life satisfaction in patients with depression (Berking et al., 2013; Ehret et al., 2014; Weytens et al., 2014), and decrease short- and long-term negative emotions in healthy individuals (Denny and Ochsner, 2014). Enhancement of self-reported CR frequency after CR training compared to the wait-list control condition (WLC) could reduce the depressive symptom severity of depressed patients (Berking et al., 2019). The change in CR tendency after CBT could predict a decrease in depressive symptoms in patients with depressive disorder (Forkmann et al., 2014). A meta-analysis of emotion regulation intervention in clinical populations showed improvement in emotion regulation (including CR) tendencies (Moore et al., 2022). However, studies of the antidepressant effect of CR training on cognitively vulnerable individuals with depression are still lacking. Yuan et al. (2014) proposed that the use of CR training could reduce the vulnerability of adolescents to negative emotions, whose development stage is prone to emotional disturbances. Thus, empirical evidence for the training effects of CR on cognitively vulnerable individuals is still needed.

Notably, the neural mechanism of CR training effects is still unclear. First, deficits in CR for depressed individuals are related to aberrant activities in frontal cognitive control regions, which modulate the emotion generation regions (Khodadadifar et al., 2022), and abnormalities in the structural covariance of the emotion regulation network (Wu X. et al., 2017). (1) Cognitive control regions: The dorsolateral prefrontal lobe (dlPFC) and the ventrolateral prefrontal lobe (vlPFC) are responsible for storing and choosing appropriate CR strategies, respectively (Morawetz et al., 2016). The dorsal medial prefrontal lobe (dmPFC) is mainly responsible for monitoring and reflecting changes in an emotional state, modifying the initial emotion appraisal in adaptation to current situations/goals (top-down control of emotion) (Phillips et al., 2003). (2) Emotion generation regions, the ventral medial prefrontal cortex (vmPFC), are mainly responsible for the initial appraisal of emotional stimuli and spontaneous emotional responses (Phillips et al., 2003). The amygdala plays a major role in the effective relevance (valence) of positive and negative stimuli (Fadok et al., 2018), and its volume (especially the right side) is positively associated with CR ability (Hermann et al., 2014). The functional interactions between prefrontal subregions (dlPFC/vlPFC/dmPFC) and the amygdala probably act through the vmPFC, which has direct structural connections with prefrontal subregions and with the amygdala (D’Arbeloff et al., 2018). When depressed patients regulated negative emotion using CR, lower activation of the dlPFC, the vlPFC, and the dmPFC and higher activation of the amygdala and the vmPFC were found during the downregulation of negative emotion, and higher activation of the medial prefrontal cortex (mPFC) and lower activation of the amygdala were found during the upregulation of negative emotion (Belden et al., 2015; Picó-Pérez et al., 2017; Stephanou et al., 2017; Zilverstand et al., 2017; Radke et al., 2018). In contrast, the IFG and dlPFC/dmPFC/vmPFC effective functional connectivity (FC) patterns are indicative of reappraisal effectiveness (RE) (Morawetz et al., 2017), and the hypoactivity of the IFG in patients with depression modulated by motivation disposition during CR may interfere with inhibitory IFG-dlPFC coupling (Wang et al., 2014, 2017).

Second, there were fewer evidence indicating neural abnormalities underlying the CR abilities of individuals vulnerable to depression. Previous evidence indicated strengthened dmPFC-amygdala and vmPFC-amygdala resting-state functional connectivities in stress-vulnerable individuals with high harm avoidance (HA) personality traits (Baeken et al., 2014). In healthy individuals, stronger coupling of the dmPFC-amygdala under task-induced stress showed an enhanced immune response (Muscatell et al., 2015), which may suggest stress vulnerability. Furthermore, the associations between lower left-lateralized vlPFC activation and decreased RI ability (e.g., RIT-fluency) of individuals with depressive symptoms and chronic stress were observed, supporting the deficits in reappraisal inventiveness in individuals vulnerable to depression (Papousek et al., 2017; Perchtold et al., 2017). In contrast, the inverse relationship between the functional activities of vmPFC and amygdala in stress-resilient individuals showed more effective control over emotion response (decreased versus increased activation of amygdala) when confronted with repeated task-induced stress compared to stress-vulnerable individuals (Wang et al., 2013).

In summary, this evidence may suggest abnormalities in the prefrontal–amygdala circuits for individuals cognitively vulnerable to depression, which may lead to heightened initial affect appraisal (vmPFC↑, vmPFC-amygdala coupling↓), failure to store, generate, and monitor the need for implementing CR strategies (dlPFC/vlPFC/dmPFC↓, dlPFC/vlPFC/dmPFC-amygdala coupling↑), and selection of appropriate contextual CR strategies (IFG↓, IFG-dlPFC coupling↓).

On the other hand, the frequent user of CR showed stronger downregulation of amygdala activation to negative emotion stimuli (Kanske et al., 2012), and CR in healthy individuals can attenuate the excitatory connectivity from the dlPFC to the inferior frontal gyrus (IFG) and increase the inhibitory connectivity from the IFG to the dlPFC, which supports the selection of appropriate alternative CR strategies (reinterpretations) in the IFG, and afterward, inhibition of original interpretations maintained and monitored in the dlPFC (Morawetz et al., 2016). Neurofeedback-guided CR training targeting the left vlPFC improved the frequency of CR strategy use (indexed with ERQ) as well as depression symptoms (Keller et al., 2021). Therefore, CR training may enhance CR ability by changing the functional activity/connectivity pattern of prefrontal-amygdala circuits, which then alleviates cognitive biases and thus depressive symptoms.

**FIGURE 1:**
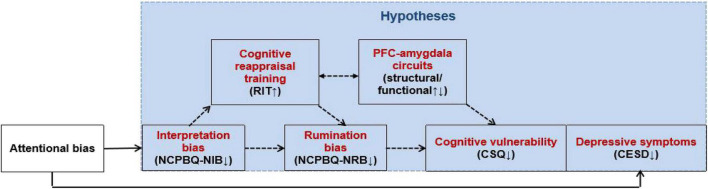
Antidepressant mechanism of CR training on individuals cognitively vulnerable to depression.

Researchers suggested that specific measures using lab-based tasks may be more sensitive to detect training effects (Cohen and Ochsner, 2018). Therefore, in this study, we will combine the RIT, CR training, and fMRI, as well as self-report measures (cognitive/emotion/symptoms) to address the following aims: (1) to examine whether the CR ability (reappraisal inventiveness) of the population with cognitive vulnerability to depression is abnormal; (2) to test the hypothesis that CR training alleviates depressive symptoms through the mediators of cognitive bias (e.g., interpretation bias and/or rumination bias); (3) to investigate the neural substrates (structural/functional) of prefrontal–amygdala circuits through which CR training improves the ability to generate a CR strategy; and (4) to examine the short- and/or long-term effects of CR training on reducing depression symptoms in cognitively vulnerable individuals immediately after intervention and/or 6 months to provide theoretical and practical evidence for early intervention in these populations. We postulate that CR training improves CR ability (RIT) by changing the brain activity/function connectivity of the prefrontal-amygdala circuits, thereby reducing negative cognitive biases (NCPBQ-NIB, NCPBQ-NRB, and CSQ) and improving depression symptoms (CESD) (Figure 1).

## Methods

**FIGURE 2:**
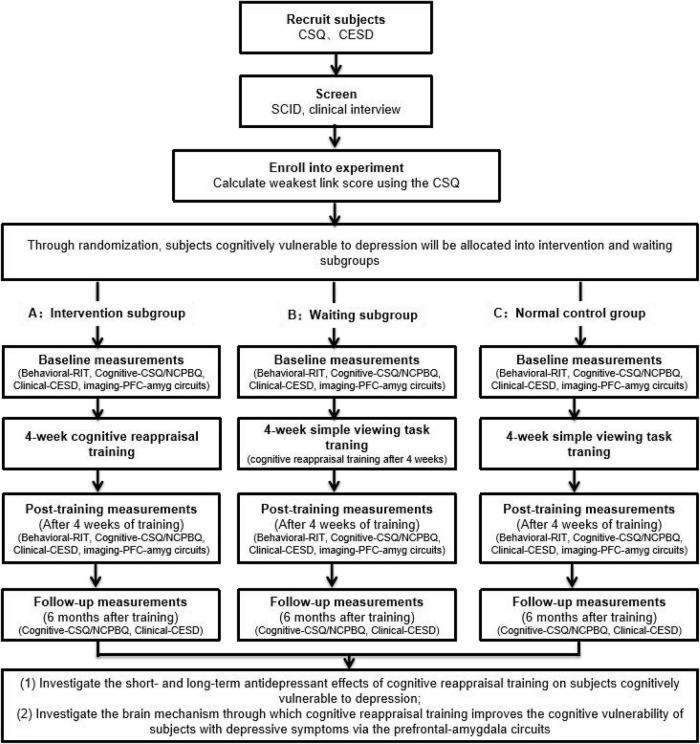
The flow diagram of the current study summarizing the randomized controlled trial design, recruitment of participants, and collected measures.

Using RIT and CR training in combination with fMRI, we will explore (1) the deficits in CR ability (indexed with RIT) for the depression-vulnerable group; (2) the short- and long-term antidepressant effects of CR training on depression-vulnerable populations; (3) whether it is feasible to reduce negative cognitive biases (negative interpretation bias and/or negative rumination bias) and depressive symptoms of depression by improving reappraisal inventiveness; and (4) the changes in the prefrontal–amygdala circuit induced by CR training, including changes in the functional activation and connectivity of the regions of interest (prefrontal cortex and amygdala) before and shortly after CR training and changes in the volumes of these regions of interest (prefrontal cortex and amygdala) long after CR training (6 months later) (Figure 2).

### Procedure

#### The effects of cognitive reappraisal inventiveness on individuals cognitively vulnerable to depression

Individuals cognitively vulnerable to depression will be screened using the Cognitive Style Questionnaire (CSQ) developed based on the hopelessness theory and the weakest link method. The weakest link method (Abela and Sarin, 2002) will be used to screen individuals with cognitive vulnerability to depression; it showed the value in predicting changes in depression (Reilly et al., 2012). By using a group of individuals cognitively vulnerable to depression and a normal control group as research participants, we will determine, using the RIT, whether individuals cognitively vulnerable to depression have defects in CR inventiveness before training.

#### Neural mechanism of improvements in cognitive reappraisal inventiveness in individuals cognitively vulnerable to depression through cognitive reappraisal training

Using the group of individuals cognitively vulnerable to depression (divided into intervention and waiting subgroups) and the normal control group as research participants, we will conduct a 4-week (2 sessions/week) CR training program for the intervention subgroup, and before and after training, the intervention and waiting-list control subgroups will undergo fMRI to examine whether CR training can improve reappraisal inventiveness and depression symptoms by regulating the function of the prefrontal–amygdala circuit.

#### Short- and long-term antidepressant effects of cognitive reappraisal training on individuals cognitively vulnerable to depression

Through follow-up immediately after intervention or 6 months after the intervention, we will validate the hypothesis of the reduction in cognitive vulnerability to depression and depression symptoms through improvements in reappraisal inventiveness with CR training by constructing a hierarchical linear model (HLM) analysis and mediating/modulating effect analyses.

### Subjects

The study protocol is in accordance with the Declaration of Helsinki and was approved by the ethical committee of the University. All voluntary participants have to provide informed written consent before the initiation of the study procedures.

#### Group of individuals cognitively vulnerable to depression

Of the recruited healthy subjects, those with an average weakest link score plus 1 standard deviation will be assigned to the group of individuals cognitively vulnerable to depression, and whether they have affective disorders and other axis I diseases will be determined by two experienced psychiatrists using the Structured Clinical Interview for DSM Disorders (SCID).

The inclusion criteria are as follows: participants (a) with average CSQ weakest link score plus 1 standard deviation; (b) with Han ethnicity; (c) aged 18–25 years; (d) who are right-handed; (e) with normal or corrected vision; (f) who are local community residents; (g) who can understand the content of the study, are willing to participate, and are committed to completing the entire experiment; (h) have signed informed consent form.

The weakest link composite score of CSQ will be calculated as follows: (1) calculating the scores for the 3 subscales (causes, consequences, and self-worth) of CSQ; (2) standardizing the scores for the 3 subscales and converting them to standard scores, based on which the standard scores for the 3 subscales for each individual are then ranked, in which the highest of the 3 subscale scores represents the final score for each individual’s weakest link; and (3) calculating the average weakest link scores for all individuals. The score is further used to screen individuals in the cognitively vulnerable to depression group (mean score plus 1 standard derivation) or those in the not cognitively vulnerable to depression group (the mean score minus 1 standard derivation).

The exclusion criteria are as follows: participants (a) who meet the diagnostic criteria for depression or other axis diseases; (b) with a history of taking any antidepressants or antipsychotics; (c) with a history of alcohol or drug abuse or dependence; (d) with a history of central nervous system diseases, e.g., head injury or epilepsy; (f) with a history of multiple sclerosis or major physical diseases; (g) with a history of electroshock therapy; (h) with a history of mental illness in first-degree relatives; and (i) with contraindications to MRI.

#### Normal control group

Of the recruited healthy subjects, those with an average weakest link score minus 1 standard deviation will compose the group of individuals without cognitive vulnerability to depression, i.e., the normal control group. Individuals in the normal control group will be interviewed by two experienced psychiatrists using the SCID to exclude those suffering from depression and other axis I diseases. Ultimately, the control group will comprise 30 subjects, and the control group and the intervention and waiting subgroups of the group of individuals cognitively vulnerable to depression will be homogenous in terms of demographic variables and depression symptoms.

The inclusion criteria are described as follows: participants (a) with average CSQ weakest link score minus 1 standard deviation; (b) with Han ethnicity; (c) aged 18–25 years; (d) who are right-handed; (e) who have normal or corrected vision; (f) who are local community residents; (g) who can understand the content of the study, are willing to participate, and are committed to completing the entire experiment; and (h) who signed an informed consent form.

The exclusion criteria are described as follows: participants (a) who meet the diagnostic criteria for depression or other axis diseases; (b) history of taking any antidepressants or antipsychotics; (c) history of alcohol or drug abuse or dependence; (d) history of central nervous system diseases, e.g., head injury or epilepsy; (e) history of multiple sclerosis or major physical diseases; (f) history of electroshock therapy; (g) history of mental illness in first-degree relatives; and (h) contraindications to MRI.

Sample size calculation: An *a priori* power analysis found that a sample size of 75 participants per group (*N* = 150) would be sufficient with a medium effect size (Cohen’s d = 0.0.39) and 65% power (computed with G*power 3.1). These estimates were based on previous research examining changes in HRSD/BDI-II scores before and after CR training when compared to the waiting-list control condition (Berking et al., 2019). Since the sample size projection considered an anticipated 20% dropout rate, the target sample size was 188 patients.

Randomization to treatment will be completed with a computer-generated list of random numbers, and each participant will be randomly assigned to either the intervention group or the control group with a 1:1 allocation and double-blinded. The randomization will minimize group differences in demographic factors and instrument scores (Pocock and Simon, 1975).

### Cognitive reappraisal training

#### Stimuli

After preliminary experiments, a total of 48 positive and negative emotion pictures were selected from the Chinese Affective Picture System (CAPS), which showed high internal consistencies of valence, arousal, and dominance (0.982, 0.979, and 0.980) (Bai et al., 2005). A block design will be adopted, in which each task block contains one type of CR task, with the same number of positive and negative emotion pictures with comparable arousal.

#### Task

The CR task for each participant will include 3 conditions: simple viewing (baseline), CR + (emotion upregulation), and CR- (emotion downregulation). The CR task starts with the baseline condition, and then the order of CR + and CR- tasks will be counterbalanced across the participants. The task was designed based on our previous study of the CR ability of patients diagnosed with depressive disorder (Wang et al., 2014, 2017). To motivate the participants to generate reappraisal strategies, the practice before the formal CR task will give the participants two examples that provide creative reappraisal strategies vs. objective descriptions of the emotion stimuli pictures (Wu X. et al., 2017, Wu et al., 2022) and then require the participants to reappraise each of the four emotion pictures (2 for the emotion upregulation condition and 2 for the emotion downregulation condition See details below) and type their strategies in the blank under each picture on the computer screen.

##### Simple viewing task (baseline)

The participants will be instructed to naturally view the emotion stimuli pictures in sequence and rate their emotional arousal after each picture. The following instructions will be provided at the beginning of the task: “When a fixation point ‘ + ’ appears in the center of the screen, please keep your eyes on the fixation point. When the fixation point disappears, a picture appears; please look at the picture. After the picture disappears, please evaluate your emotional intensity at this time based on the instructions provided.”

##### Cognitive reappraisal + task (emotion upregulation)

The participants will be instructed to enhance their emotions while viewing stimuli pictures and rate their emotional arousal after each picture. The following instructions will be provided at the beginning of the task: “When a fixation point ‘ + ’ appears in the center of the screen, please keep your eyes on the fixation point. When the fixation point disappears, a picture will appear. Please imagine yourself in the picture and fully experience the emotion the scene evokes. After the picture disappears, please evaluate your emotional intensity at this time based on the instructions provided.”

##### Cognitive reappraisal-task (emotion downregulation)

**FIGURE 3:**
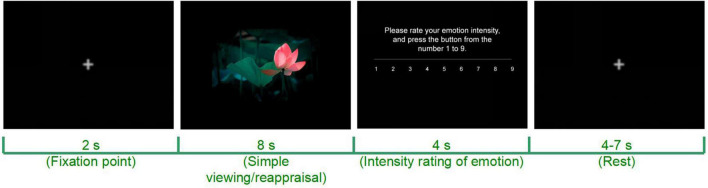
Schematic diagram of a computerized CR task. Adapted from https://699pic.com/.

The participants will be instructed to weaken their emotions while viewing stimulus pictures and rate their emotional arousal after each picture. The following instructions will be provided at the beginning of the task: “When a fixation point ‘ + ’ appears in the center of the screen, please keep your eyes on the fixation point. When the fixation point disappears, a picture will appear. Please keep an objective attitude and try not to feel any emotion from the picture. After the picture disappears, please evaluate your emotional intensity at this time based on the instructions provided” (Figure 3).

The emotional regulation effectiveness of CR can be calculated by subtracting the emotional intensity for the simple viewing task from that for the CR + task or CR- task. The upregulation effect (arousal_*CR*+_ - arousal_*baseline*_) and the downregulation effect (arousal_*baseline*_ - arousal_*CR*–_) can be obtained before (T_0_) and after (T_1_) CR training while MRI is performed (T_0_ and T_1_).

#### Training procedure

**FIGURE 4:**
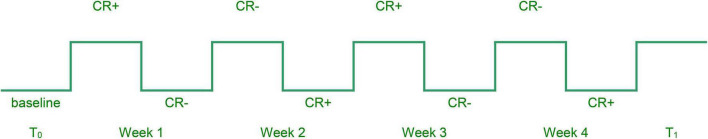
CR training process: one session as an example (4 weeks, 2 sessions/week).

The training includes 8 sessions (2 sessions/week) within 4 weeks. Each session is divided into 9 blocks (20 trials/block, 180 trials in total). The order of the experimental content is counterbalanced, i.e., in each block, one-half of the subjects start with a CR + task and the other half start with a CR- task. In each session, pictures with different valences are randomly presented in trials. Each trial lasts 18–21 s: first, a fixation point is presented for 2 s at the center of the screen which the participant fixes; then, a positive or negative emotion picture is presented on the screen and the subject is asked to focus on the emotion picture for 8 s to allow emotional arousal. After the picture disappears, the subject is asked to report his/her emotional intensity at the moment on a 1–9 scale within 4–7 s, during which the subject’s reaction time of key press is recorded. If the subject does not press the key in the allotted time, the interface screen will continue (Figure 4).

After the experiment, the subject is asked about his/her conformity to the instructions (manipulation check). Both the quantitative and qualitative CR manipulation checks will be conducted by the experimenter to confirm that they obeyed CR training instructions (Rood et al., 2012). After each block, participants briefly state the content coming into mind during that block (recorded by the experimenter after the informed written consent by the participants) and the degree to which they reappraise the emotional pictures during that block on a 0–100 rating scale.

### Primary outcome

The structured clinical interview for DSM-IV-TR Axis Disorders (SCID-I) will be used to diagnose and screen for major depressive disorders and exclude other DSM-IV axis I mental disorders when recruiting subjects. Demographic information, including gender, age, ethnicity, education, and duration of illness (months), will be collected using a questionnaire.

#### Center for epidemiologic studies depression scale

The center for epidemiologic studies depression scale (CESD) will be used by subjects to self-evaluate their current depressive symptoms (within a week), focusing on depressive emotions or mood rather than somatic symptoms. Subjects with depressive symptoms will undergo preliminary screening to facilitate further diagnosis. The scale contains 20 items in 4 dimensions (negative emotions, positive emotions, physical symptoms, and interpersonal relationships) that are scored on a 4-level scale (“no or rarely, less than once a day,” “sometimes or only a small part of the time in 1 day,” “frequently or half of the time in 1 day,” and “most of the time or all the time in 1 day”); the higher the score is, the more severe the level of depression.

### Secondary outcomes

#### Negative cognitive bias

##### Cognitive style questionnaire

The CSQ will be used to screen subjects in the group of individuals cognitively vulnerable to depression and the group of individuals not cognitively vulnerable to depression. The CSQ was developed based on the hopelessness theory and was designed to be used to assess an individual’s negative cognitive styles, mainly including the individual’s perceptions of the causes, consequences, and self-worth after an event. The Chinese-translated version of the CSQ has good internal reliability (Cronbach’s alpha coefficient = 0.94), satisfactory 8-week test–retest reliability (reliability = 0.59), and good construct validity for the four-factor model (CFI = 0.91, RMSEA = 0.09), which included general-local causes, stable-temporary causes, consequences, and self-worth (Chen et al., 2014).

##### Negative cognitive processing bias questionnaire

The negative cognitive processing bias questionnaire (NCPBQ) contains 4 dimensions, i.e., negative attention bias (NAB), negative memory bias (NMB), negative interpretation bias (NIB), and negative rumination bias (NRB), using a Likert 4-point scale from 1 (completely disagree) to 4 (completely agree). The NCPBQ has good internal reliability (Cronbach’s alpha coefficient = 0.89), split-half reliability (*r* = 0.87), construct validity for a four-factor model that explained 50.152% of the total variation (CFI = 0.92, RMSEA = 0.05), and criterion validity coefficients of 0.395 (relative to the Beck Depression Self-Rating Scale) and 0.548 (relative to the Dysfunctional Attitude Questionnaire) (Yan et al., 2017). The negative interpretation bias (NIB) and the negative rumination bias (NRB) subscales will be utilized in the mediation analyses.

#### Reappraisal ability/tendency

##### Reappraisal inventiveness test

RIT includes 4 scenes that can trigger emotions. Participants are asked to imagine themselves in the scene as vividly as possible within a specified time (3 min), after which they are asked to think of reappraisals that can change the induced emotion within 20 s and then record the reappraisals in 3 min. RIT was scored on two scales: (a) RIT-fluency was indicated by the number of reappraisals generated and (b) RIT-flexibility was indicated by the number of categories of different reappraisals (Weber et al., 2013). To improve the internal validity of the test, we set the induced emotions to sad and happy. Before the test, the subjects will be given examples to illustrate how to generate as many reappraisals as possible for a given emotional stimulus so that they can transfer to the formal test.

##### Emotion regulation questionnaire

The ERQ was developed by Gross and contains a total of 10 items in 2 dimensions, a CR and expression inhibition (EI) (Gross and John, 2003). It is scored through a 5-point scale (1 = strongly disagree and 5 = strongly agree); the higher the score, the more likely the subject is to use this emotion regulation strategy. The Chinese-translated version of the ERQ has good test–retest reliability (r = 0.82 and 0.79) and internal reliability (Cronbach’s alpha coefficient = 0.85 and 0.77) for the CR and EI subscales, split-half reliability (reliability = 0.87), and construct validity for the two-factor model (CFI = 0.96, RMSEA = 0.09), with completely standardized factor loadings of all items higher than 0. 55 at a statistically significant level (Wang et al., 2007). The ERQ will be utilized to verify the construct validity of the RIT indexes.

### Brain structure and functional imaging

Brain structure and functional imaging data will be collected on a Siemens 3.0T scanner from the affiliated hospital of the university. During the scan, each participant will be in a supine position, with foam under the head to minimize head movement, and will be wearing headphones to reduce noise interference. A standard orthogonal single-channel head coil will be used. Conventional T1WI and T2WI imaging, echo-planar imaging (EPI), and whole-brain 3D magnetization-prepared rapid gradient-echo (MPRAGE) imaging will be conducted for each subject by a radiologist. The experimental stimulus will be presented by connecting a liquid-crystal display (LCD) screen outside the scanning channel to the E-prime stimulus presentation and the response recording system, and each participant will view the presentation through a reflective mirror attached to the head coil. During the experiment, the participant will use a joystick to enter their ratings while the system automatically records the participant’s response to the CR tasks.

The scanning sequence will be as follows: **(1)** The axial T1-FLAIR sequence, parallel to the anterior-posterior joint line; pulse repetition time/echo time (TR/TE) = 500 ms/14 ms; slice thickness = 4 mm; field of view (FOV) = 240 mm × 240 mm; and matrix = 256 × 160; **(2)** sagittal T2-FLAIR will be scanned at the same location using gradient-echo echo-planar imaging (EPI) with the following parameters: TR/TE = 3,000 ms/40 ms; FOV = 240 mm × 240 mm; matrix = 128 × 128; 30 consecutive images at each time point; and slice thickness = 4 mm; if no abnormality is observed in plain scans, resting-state scans will be performed; **(3)** a total of 176 consecutive slices in the sagittal position will be acquired to cover the entire brain through an MPRAGE sequence for subsequent 3D reconstruction and spatial alignment with the following parameters: TR/TE = 1,970 ms/3.93 ms; flip angle = 15°; slice thickness = 1.70 mm; interval = 0.85 mm; FOV = 250 mm × 250 mm; and matrix = 448 × 512; and **(4)** task-state blood-oxygen-level-dependent (BOLD) functional images will be acquired using the EPI sequence, with the same position as that for anatomical images and the following parameters: TR/TE = 2,000 ms/30 ms; number of slices = 36; slice spacing = 33; FOV = 240 mm × 240 mm; and matrix = 64 × 64.

Regions of interest (ROIs): The structural and functional activities of the dlPFC, the vlPFC, the dmPFC, and the vmPFC, as well as the amygdala, will be analyzed. The functional connectivities between these regions and subcortical regions (amygdala) will be analyzed based on the time series of these ROIs.

### Statistical analysis

#### Behavioral and self-report data

##### Intervention effect analysis

A 3 (group: CR = cognitively vulnerable intervention subgroup; WC = cognitively vulnerable waiting-list control subgroup; NC = normal control group) × 3 (time point: T_0_ = pretraining, T_1_ = post-training, T_2_ = 6-month follow-up) mixed design will be adopted. Using the HLM analysis, as well as mediating/modulating effect analyses, we will investigate (1) hypothesis 1, whether the changes in negative cognitive biases and depressive symptoms across time 1 and time 2 differ significantly among groups and (2) hypothesis 2, whether the changes in negative cognitive bias (negative interpretation bias and/or negative rumination bias) pre- and post-training could predict depression symptoms at T_1_ and/or T_2_.

To test hypothesis 1, we will conduct an HLM analysis with time (0 = pretraining; 1 = post-training), group (0 = CR; 1 = WC; 2 = NC), and the interaction between time and group as predictor variables and the with CESD score as the outcome variable. To test hypothesis 2, we will conduct mediating/modulating effect analyses among CR ability (RIT) and tendency (ERQ), negative cognitive bias (NCPBQ), cognitive vulnerability (CSQ), and depressive symptoms (CESD) at T_1_ and/or T_2_. The HLM and mediating/modulating effect analyses will be conducted in SPSS (Statistical Product and Service Solutions) software version 22.0 (SPSS, Inc., Chicago, IL, United States) combined with the PROCESS Macro (Hayes, 2012).

#### Neuroimaging data

##### Data preprocessing

Structural data preprocessing: The raw data will be analyzed with the CAT (Computational Anatomy Toolbox) 12 toolbox.^1^ (1) Segmentation: The T1-weighted image for each pair of participants will be normalized to a template MNI space using DARTEL normalization and segmented into gray matter (GM), white matter (WM), and cerebrospinal fluid (CSF). (2) Check data quality: The written data will be checked to exclude those with artifacts or orientation errors. (3) The GM images will be smoothed using a Gaussian kernel of 8 mm full-width half-height (FWHM) and then entered into the statistical analysis.

Functional data preprocessing: The raw data will be formatted using MRIcro software^2^ and the data will be preprocessed using the internationally accepted software Statistical Parametric Mapping 12 (SPM12) (Wellcome Trust Center for Neuroimaging.^3^ (1) Functional data for the first 10 repetition times (TRs) will be excluded to eliminate the effects of subject maladaptation and longitudinal magnetization relaxation not reaching the steady state. (2) Head movement correction: This will be performed to reduce the interference of noise signals caused by the subject’s head movement during the scanning process, and data with head motion exceeding 1.5 mm or a rotation angle exceeding 1.5° will be excluded. (3) Time point correction: This will be performed to standardize the images collected at different time points to the same time point based on the time modulation effect of the hemodynamic function on each functional image. (4) Alignment: To address the differences in the anatomical structure of different individuals, each subject’s functional images and templates will be aligned so that the individual’s brain is aligned to the standard Montreal Neurological Institute (MNI) space for functional localization of activation zones. (5) Gaussian smoothing: The full width at half maximum value will be set to 8 mm to improve the signal-to-noise ratio of images.

##### Data analysis

(1) Analysis of the morphological characteristics of brain structure: Voxel-based morphometry (VBM) will be adopted to calculate the concentration and volume of each voxel in different brain tissues (gray matter, white matter, and cerebrospinal fluid) using templates for the whole brain and different brain tissues created based on 3D data, and the volumes of ROIs will be compared between different groups to examine (1) whether the volumes of ROIs before and after CR training differ significantly and (2) if the volumes of ROIs differ significantly, whether the changes in the volumes of the ROIs can predict depressive symptoms immediately after training (T_1_) or 6 months later (T_2_).

(2) Region of interest analysis: The anatomical localization of functional images will be determined with reference to the anatomical maps of the brain in Talairach coordinates and based on the opinions of experienced neuroimagers. Using MARSbar software, the prefrontal subregions (dlPFC/dmPFC/vlPFC/vmPFC) and the amygdala will be extracted as BOLD signals of ROIs by subtraction between the baseline and CR (±) conditions. The data will then be exported to SPSS for statistical processing, in which the activation of the region of interest will be used as a predictor of depressive symptom scores to investigate (1) whether the activation levels of regions of interest (prefrontal lobe and the amygdala and its subregions) before and after CR training differ significantly and (2) if the activation levels do differ significantly, whether the changes in the activation levels of the regions of interest can predict depressive symptoms (T_1_ and T_2_).

(3) Functional connectivity analysis: Taking the amygdala as the seed point and the average time series within the seed point as the reference time series, we will perform “one point to multipoint” linear correlation analysis with the time series of each ROI. Using the average signals of head movement, the white matter, the cerebrospinal fluid, and the whole brain as covariates and using the prefrontal-amygdala FCs as predictors of depressive symptom scores, we will use between-group *t*-tests, combined with correlation analyses and path analyses, to examine (1) whether the FCs between the prefrontal subregions (dlPFC/vlPFC/dmPFC/vmPFC) and the amygdala before and after CR training differ significantly and (2) if the prefrontal-amygdala FCs do differ significantly, whether the FCs could predict depressive symptoms (T_1_ and T_2_). The FCs between ROIs, cognitive bias (CSQ/NCPBQ), CR ability (RIT), and tendency (ERQ) will then be entered into path analyses to verify the cognitive bias model of CR training.

## Discussion

CR is one of the key components of CBT, and accumulating evidence suggests that CR training as a standalone treatment is effective for depressed patients to improve negative effects and enhance wellbeing (Berking et al., 2013; Ehret et al., 2014; Weytens et al., 2014). Prior studies found that individuals with cognitive vulnerability are at high risk for depression, which merits attention considering the prevalence of depression. Nonetheless, as a highly accessible and cost-effective intervention, the mood-lifting effects of CR for cognitively vulnerable individuals are unknown. Therefore, the current study could provide support for the potential of CR training for the prevention of depression in vulnerable groups. Of note, with the progress of e-mental health, CR training is promising to be developed into a cost-effective computerized program that is easy to implement for counselors and is suitable for early interventions of cognitively vulnerable individuals to depression.

Furthermore, despite ample evidence for the role of the prefrontal–amygdala circuit in CR, as well as prefrontal–amygdala dysfunction in CR deficits in patients with depression, the impact of CR training on hypothesized neural targets remains unclear, especially the cause–effect relationship between these regions during CR training. Based on previous evidence, we propose that CR training may involve increased top-down cognitive control areas (such as dlPFC, vlPFC, and dmPFC) and reduced bottom-up emotion responding areas (such as vmPFC and amygdala), as well as strengthened interactions between the PFC and the subcortical regions (mainly the amygdala). In addition, the hypothesis about the changes after CR training is exploratory and without strong *a priori* evidence; however, the structural changes of CR training may be of clinical significance. The current study aims to reveal the neural substrates of CR training, which remain largely unknown yet and have the potential to inform techniques, such as real-time fMRI neurofeedback (Zweerings et al., 2020; Keller et al., 2021), to precisely impact the neural targets underlying CR and amplify the effects of CR training.

This study also proposes a novel cognitive bias model of CR training (CR training→cognitive bias↓→cognitive vulnerability to depression↓), which hypothesizes that CR training may improve CR ability (effectiveness and incentives) and reduce cognitive bias (negative interpretation bias and/or negative rumination bias), which then alleviates the depressive symptoms. We adopt a process-based approach, which emphasizes the primary cognitive process underlying intervention (Forgeard et al., 2011) and assesses the effectiveness of the training using behavioral (RIT), cognitive (CSQ/NCPBQ), and clinical (CESD) assessments, along with measures of task-related brain activity/FCs before and after the training. This approach may provide proof-of-concept evidence for the cognitive bias model of CR training. In summary, the following scientific questions are expected to be answered: whether CR training will change prefrontal–amygdala FCs in the short term and the structure of the regions among the circuits in the long term (6 months later). The current study is promising to provide a novel approach to the intervention of depression vulnerability and insights into the underlying neurocognitive mechanism.

## Limitations

First, we include the process measures of cognitive bias (negative interpretation bias and/or negative rumination bias) to evaluate the intervention effects, which should be interpreted as exploratory. More solid progress will depend on a systematic examination of the neural network mechanism involved in CR training effectiveness. The neural signature of the cognitive control and emotion regulation network during CR, such as global metrics and topological characteristics (Pan et al., 2018), is needed. However, connectome-based predictive modeling of distributed neural networks during CR has not revealed a meaningful whole-brain signature of CR tendency (Pan et al., 2018; Burr et al., 2020). Therefore, more evidence is needed to explore the neural network mechanism of CR training, which may provide more perspectives on the neural and cognitive targets of CR training.

Second, evidence of both clinical effectiveness and cost effectiveness of CR training is necessary for future studies to test the feasibility of CR training in primary care, as well as in clinical settings for vulnerable individuals with depression, and generalize CR training to those populations that may benefit from training. Therefore, various context-specific CR training programs should be forged into well-specified protocols that could be tested in randomized controlled trials to provide such evidence. Furthermore, further studies comparing CR training with other components of CBT as well as its whole package are warranted to assess its cost-effectiveness.

## Conclusion

This study is expected to provide initial evidence on the intervention effect of CR training on cognitively vulnerable individuals with depression through the mediating role of cognitive bias (negative interpretation bias and/or negative rumination bias) with the underlying prefrontal–amygdala structural/functional neural substrates.

## Ethics statement

The studies involving human participants were reviewed and approved by the Ethics Committee of Third Military Medical University. Written informed consent was not provided because no participant has been recruited and/or included in the study at the time of submission.

## Author contributions

XW and ZF conceived and designed the study. XW drafted the manuscript. All authors revised the manuscript, approved the final version of the manuscript, and agreed to authorship contributions.

## Footnotes

1
https://neuro-jena.github.io/cat/

2
http://www.mricro.com

3
http://www.fil.ion.ucl.ac.uk/spm/software/spm12/
